# 3Rs Principle and Legislative Decrees to Achieve High Standard of Animal Research

**DOI:** 10.3390/ani13020277

**Published:** 2023-01-13

**Authors:** Paolo Verderio, Mara Lecchi, Chiara Maura Ciniselli, Bjorn Shishmani, Giovanni Apolone, Giacomo Manenti

**Affiliations:** 1 Unit of Bioinformatics and Biostatistics, Fondazione IRCCS Istituto Nazionale dei Tumori, 20133 Milan, Italy; 2 Scientific Directorate, Fondazione IRCCS Istituto Nazionale dei Tumori, 20133 Milan, Italy; 3 Unit of Animal Health and Welfare, Fondazione IRCCS Istituto Nazionale dei Tumori, 20133 Milan, Italy

**Keywords:** 3Rs principle, replacement, reduction, refinement, in vivo experiments, animal welfare

## Abstract

**Simple Summary:**

The 3Rs principle refers to three concepts: Replacement, Reduction and Refinement. These principles should be taken into consideration during the planning and execution of experiments by trying to replace the animal model with an alternative model (if possible), reduce the number of animals by adopting proper and efficient statistical designs, and refine and improve the experimental conditions. The first application of this principle in Europe was reported in the document of the Council Directive of 24 November 1986 (86/609/EEC), and then developed in the updated Directive 2010/63/EU of the European Parliament and of the Council of 22 September 2010. Here, we discuss the perspectives of the 3Rs principle, with a particular focus on the concept of Reduction.

**Abstract:**

Animal experimentation is a vast ecosystem that tries to make different issues such as legislative, ethical and scientific coexist. Research in animal experimentation has made many strides thanks to the 3Rs principle and the attached legislative decrees, but for this very reason, it needs to be evenly implemented both among the countries that have adhered to the decrees and among the team members who design and execute the experimental practice. In this article, we emphasize the importance of the 3Rs principle’s application, with a particular focus on the concept of Reduction and related key aspects that can best be handled with the contribution of experts from different fields.

## 1. Introduction

Although about 60 years have passed since its publication, the 3Rs principle has not lost its relevance; indeed, the ideas put forward by Russell and Burch [1] are still current and continue to represent a flexible and valuable tool to guarantee both the welfare of the animals used in laboratories and the quality of the data collected. The 3Rs principle refers to three concepts: Replacement, Reduction and Refinement. During the planning phase and the execution of an in vivo experiment, the researcher should follow this principle as much as possible, trying to (i) replace, where possible, the animal model with an alternative model, (ii) reduce as much as possible the number of animals used by adopting experimental designs that are as efficient as possible and (iii) refine and improve the experimental conditions where animals are involved.

The first application of this principle in Europe was reported in the document of the Council Directive of 24 November 1986 (86/609/EEC) [2], and developed in the updated Directive 2010/63/EU of the European Parliament and of the Council of 22 September 2010 [3], which describes the issues related to the protection of animals used for scientific purposes and regulates the use of animal models in the European Union. All member states have to transpose the Directive 2010/63/EU in its legislation, and Italy must specifically follow the Legislative Decree 26/2014 [4].

Here, we discuss the perspectives of the 3Rs principle with a particular focus on the concept of Reduction and key aspects related to in vivo study implementation, such as blinding and randomization, together with statistical–methodological issues related to experimental design. It is beyond the scope of this communication to report an exhaustive description of the available statistical designs, although proper references will be provided throughout the text.

## 2. The 3Rs Principle

### 2.1. Replacement

The concept of Replacement refers to the possibility of replacing the animal model with an alternative one, thanks to the use of non-sentient material [1]. Russel and Burch [1] described a number of alternative methods, such as plants, microorganisms, bio-chemical systems and non-living physicists, and distinguished between relative and absolute replacing techniques [5]. Over time, this concept has evolved into partial replacement (i.e., relative replacement) and full replacement (i.e., absolute replacement). Partial replacement implies the use of another species characterized by a relatively less complex nervous system than the original one and that currently are not considered capable of experiencing suffering [6], such as invertebrates and immature forms of vertebrates or the use of primary cells (and tissues) taken from animals killed solely for this purpose (i.e., not having been used in a scientific procedure that causes suffering). Conversely, as regards to full replacement, in 2005, Buchanan-Smith [7] declared that the 3Rs “still remain the best approach to alternatives” and defined the concept of Replacement as “the set of procedures that completely eliminate the use of some animals”. In this case, the animal model is completely eliminated by the use of an alternative method such as tissues and cells, established cell lines and mathematical and computer models.

#### Alternative Methods

Currently, alternative methods to the animal model include the use of a variety of bioengineered in vitro and ex vivo systems, including organoids, scaffold-based 3D models and microfluidic systems to resemble and capture aspects of human physiology that were unthinkable until few years ago with a good level of realism [8]. The main feature of all these methods relies on the opportunity to address advanced question due to their more intact cytoarchitectural structures and microphysiological processes as well their multiple interacting cell types and multiorgan ones. Although these models can be interpretable by adopting conventional techniques available to pathologists, efforts for their characterization, validation and standardization are needed to confirm their adequacy for the specific question and their fully applicability [9].

For example, concerning bioprinted microtissues, some key aspects should be taken into account, including the selection of cell types and growth media as well as the composition of extracellular scaffolds that support a defined (2D or 3D) architecture [9]. Similarly, mini-organs are designed to reflect the structure and key functions of a complete organ, with a 2D or 3D tissue architecture depending on the nature of the model fabrication process [9]. A special emphasis regards organoids, which are 3D structures organized in a specific spatial pattern characterized by an organ-like architecture resembling the organ of interest and including the biological functions of those tissues without the influence of other organs and systems of the whole organism [9,10]. As recently reported by Kim et al. [11], different cancer types have already been cultured as cancer organoid models. Although cancer organoids represent a powerful and potential in vitro system for drug screening and for predicting the best therapeutic options for individual patients, additional research is needed in order to standardize the set-up of entire organoid protocols.

Already in 2013, Lancaster and colleagues [12] described so-called cerebral organoids to recapitulate the human brain tissue. Then, in 2018, Madhavan et al. [13] reported the generation of oligocortical spheroids to study the myelination of the developing central nervous system. Koike et al., in 2019 [14], developed hepato-biliary-pancreatic organoids as a model for the study of complex human endoderm organogenesis. Chen and colleagues [15] provided in their review a complete picture of the recent advancements in fabricating vascularized tissue and organs, including novel strategies and materials, and their applications. They also explored the limitations of vascularized tissue engineering and some of the promising future directions this technology may bring. Moreover, Costa et al., in 2017 [16], presented a method to produce microfluidic chips containing miniaturized vascular structures to mimic the architectures of blood flow patterns in arterial thrombosis. In the oncological area, the availability of a microfluidic system that recapitulates the physiological and pathological characteristics of human tissues and organs could, for example, open a new, interesting prospective in mimicking the tumor microenvironment (TME) and also in recapitulating the interplays between cancer and the immune system [17]. Further alternative approaches to animal use were presented by Marchesi and colleagues a few years ago [18]; they created an in vitro network representing a simplified model of the nervous and cardiovascular systems’ crosstalk. Last year, Barra et al. [19], using bioreactors, simulated the anatomical–physiological complexity of the blood–brain barrier in vitro, potentially contributing to improving the management of neurodegenerative diseases in accordance with the 3R principles. A final note concerns the organ-on-chip (OcC) platforms, a recent innovation for advanced in vitro modeling, and 3D bioprinting [20]. Despite their potential, significant challenges remain and efforts are needed in validating these technologies for biomedical research applications, also in regard to multiorgan models [20].

### 2.2. Reduction

The principle of Reduction refers to the reduction in the number of experimental units used in an experimental protocol to obtain relevant and robust results [1]. However, this does not imply a mere reduction in the number of experimental units but rather a correct planning of the experiment through the use of experimental designs suited to the objectives and the statistical nature of the variables under investigation. This presupposes the employment of the statistical theory of “Experimental Design” including issues related to the design, methods to control source of bias, and sample size determination, and therefore, it may require the involvement of experts in (bio)statistics.

Reduction can occur, for example, by using results deriving from previous studies or pilot studies planned to characterize the variables under investigation and the variability associated with them, adopting the most efficient experimental design and a sample size appropriate for the goal. Another option to reduce the number of experimental units is to harmonize the methodologies used by various laboratories/research institutions in order to promote the sharing of positive results as well as negative results. This would be of help in reducing the replication of similar experiments and/or negative results. Three levels of reduction can be classified as follows [21]:

(i) Intra-experimental reduction: This kind of reduction concerns the number of animals within each individual experiment or individual protocol within a more complex experiment. By improving the statistical design, carrying out pilot studies and possibly through a retrospective analysis of previously obtained data, it is possible to optimize the minimum number of animals to be used in the experiment in question;
(ii) Supra-experimental reduction: Here, the focus is to disseminate, as much as possible, the concept of reduction among researchers involved at various levels in animal experimentation. This can be achieved through the implementation of ad hoc courses and meetings on experimental designs or statistical methods;
(iii) Extra-experimental reduction: The reduction in this case is obtained through the evolution of experimental practice at an international level, through harmonization of the rules related to experimentation and the development of new research and testing strategies.

By focusing on the intra-experimental level, the reduction in the number of animals used in experiment derives from correct planning of the experimental design and the related statistical analysis. The use of too many EUs, besides being unethical, would lead to a waste of effort and resources in terms of money and time. At the same time, the use of an insufficient number of animals could lead to the loss of a significant result due to the lack of statistical power. Even in this case, there would be a waste of resources. The planning of the entire research flow allows one to obtain the required information and answer(s) to the original scientific question(s) with satisfactory statistical power and optimized sample size.

#### Study Design

**Figure 1:**
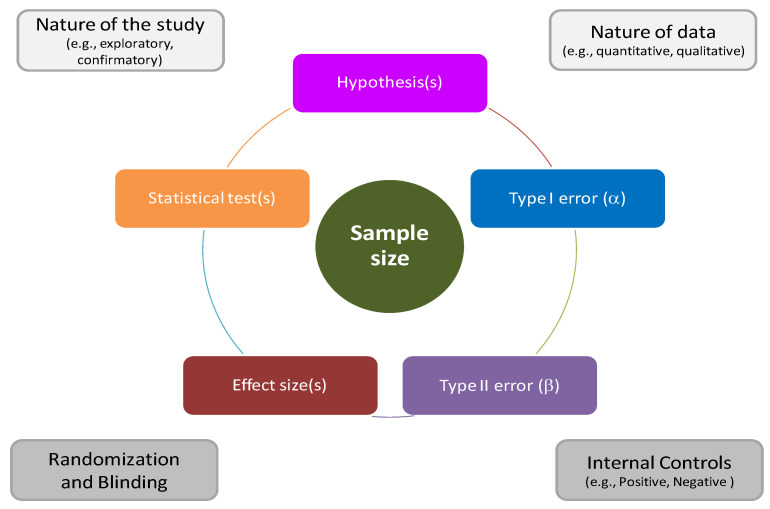
Key statistical–methodological factors to be considered in planning an experiment.

Before starting the experiment, the researcher must postulate a clear hypothesis to test and must already know how the data will be analyzed in order to avoid the unnecessary use of animals in accordance with the 3Rs. The experimenter’s question, the type of variables under consideration and, consequently, the statistical test to be applied, as well as the size of the hypothesized effect and the levels of error that one is willing to accept, will influence the design of the experiment and the number of animals necessary for its management. Figure 1 summarizes the main statistical–methodological factors to be considered in planning an experiment.

First, the design of the experiment must take into account the nature of the study, i.e., exploratory or confirmatory (Figure 1, upper part). Exploratory studies usually involve the investigation of multiple hypotheses, possibly evaluating multiple objectives [22]. These studies (also called hypothesis-generating studies) are usually used in the initial stages of research to develop new hypotheses, which can be formally tested later. Confirmatory studies, on the other hand, are designed to verify the validity of a specific hypothesis developed a priori. In this case, the statistical analysis should only be focused on the specific hypothesis, and if more statistical tests are performed, adjustments for multiple comparisons (i.e., Bonferroni correction or false discovery rate) should be adopted to reduce the risk of false positive results [22,23].

In addition to the nature of the study, the type of the involved variable(s) should be clearly defined. Quantitative data are measured on a continuous numerical scale (e.g., weight, tumor volume) or on a discrete one (e.g., number of injections, number of metastasis), whereas qualitative data can be measured on a nominal (e.g., genotype) or ordinal scale (e.g., severity score). A special case of nominal scale is represented by the binary scale, in which only two levels are available (e.g., presence/absence of a response). According to the nature of the variable(s), specific statistical models should be adopted.

Once the above-mentioned factors are defined, the next steps are represented by the formal sample size estimation (Figure 1, middle part). Briefly, researchers have to define the hypothesis system, i.e., the null (H_0_) and alternative (H_1_) hypotheses; the latter can be one-sided or two-sided. In parallel, the level of Type I and II errors should be defined: the first one represents the probability of rejecting H_0_ when it is true (i.e., false positives results—α level), whereas the second one represents the probability of not rejecting H_0_ when it is false (i.e., false negative results, β level). The complement of Type II error is called statistical power (γ = 1 − β). The last two components that should be defined—according to the nature of the variable(s)—are the statistical test to be implemented and the corresponding effect size, i.e., the smallest difference that we want to detect at the chosen α level. All these inputs are fundamental ingredients for a formal estimation of the sample size.

By moving towards the implementation of the experiments (Figure 1, lower part), it could be useful to evaluate the possibility of including appropriate experimental controls. Specifically, negative controls ensure that an unknown variable does not influence the experimental outcome; for example, animals can be treated with placebo compared to an active treatment or simulated surgery versus surgery. This could defend the researchers against false positive results. Positive controls, on the other hand, ensure that the experiment is actually able to detect the expected effect. Failure to respond in these controls could imply, for example, an experimental bias and ultimately research costs spent without obtaining clear evidence.

Finally, during study implementation, it is important to consider two key aspects: randomization and blinding [23]. The first one equalizes both measured and unmeasured confounders across treatment groups, isolating the experimental treatment as the only difference between them [24]. Moreover, it ensures that other factors, except the treatment/experimental factor under investigation, do not affect the outcome. If the outcomes of the treatment group and control group show differences, this will be the only difference between groups, leading to the conclusion that the observed difference is treatment-induced. Not only a lack of randomization but also errors related to this process are common [25]. Indeed, as summarized in Chusyd DE et al. [26], some authors report non-random allocation as random, or in other studies, animals are randomly allocated to an experimental group together but the data are analyzed as if they were randomized individually, without taking into account the intra-group correlation and ignoring the clustering. This could address misleading conclusions, finding a treatment effect when there is actually no evidence. A full awareness of the identification of the experimental unit and of the related randomization are fundamental steps to avoid recurring mistakes. As previously mentioned, randomization is the process of assigning experimental units to treatments, independently of the pre-randomization characteristics of those units, both observed and unobserved, that could confound the outcome [26]. According to Lazic et al. [27], the experimental units have to meet some conditions: (i) they must be independently randomized to the treatment conditions, (ii) they must not influence each other, especially the outcomes of interest, and (iii) the treatment must be independently applied to each EU. Notably, when animals are caged or housed in the same treatment group (i.e., not independent of each other or they could influence each other’s outcomes), the cage and not the single animal has to be considered as the experimental unit. In such a scenario, proper adjustments must be made during sample size estimation, for example, by considering the intra-class correlation (ICC) [28] or by considering the cage as an experimental unit. Especially in this case, where the sample size could be different from the number of animals [29], the involvement of biostatisticians also during the data analysis phase could be suitable in order to apply the most appropriate methods able to ensure robust and replicable results [25]. To prevent any errors, statistical support from the initial phases of the experimental planning is, in any case, strongly recommended.

As mentioned, another key aspect is blinding, which consists of removing as many prejudices of researchers as possible in evaluating the study’s measures. It implies, for example, the blindness of the researchers who perform the measurements to the treatment/condition under investigation, as well as the use of anonymized codes. Unfortunately, it is not always possible to apply it, for example, when different types of drug administration (e.g., subcutaneous, oral, intra-muscular) are under investigation. In these cases, it could be useful to involve independent technicians/researchers in the different phases of the experiments (e.g., one for treatment administration and another one for outcome’s measurement) or blind the technicians/researchers regarding some details (e.g., dose of the drug) in order to limit as much bias as possible due to unintentional prejudices regarding the treatment/experimental condition of interest.

Once all these elements are defined, and according to the objectives of the study, it will be possible to design the experiment and plan to collect the data in the appropriate way in order to answer the biological question. Inadequate experimental designs may produce biased or inconclusive answers or lack generalizability [30,31]. Without any purpose of providing a complete and exhaustive examination of all the available designs, we briefly summarize the most common ones.

In a Completely Randomized design, experimental units (e.g., mice) are assigned to different treatments at random. In this way, any significant differences between conditions can be fairly attributed to the treatment of interest by ignoring the nuisance factors that could affect treatment conditions. Instead, when a nuisance factor (e.g., reagents batch) may influence the experimental response but is not of interest and is known and controllable, experimental units with a similar nuisance factor should be grouped into blocks, leading to a Randomized Complete Block design. In this design, each treatment is randomly assigned to one experimental unit in each block. Moreover, when the interest is focused on the effect of many discrete factors (e.g., presence/absence of a treatment or different levels of a substance) on the quantitative measurement, it could be useful to implement a Factorial design. In such a design, it is possible to investigate the impact of changes of two or more factors by considering all possible combinations of the levels of each factor and also if the effect of one factor depends on the level of another (i.e., interaction). Other types of designs are the Hierarchical Nested ones that are characterized from EUs sampled multiple times—typically to obtain a more accurate measure of the EU’s response—or the Repeated Measures design, in which repeated measurements are made on each experimental unit according to a factor of interest. Finally, if assumptions under the Cross-over design are met, each EU could receive multiple treatments with a wash-out period between exposures and outcome measurement. Detailed descriptions of such designs as well as other types of designs available in the literature are reported in Sorzano COS et al. [23].

By taking into consideration all of the above mentioned issues, it is clear that proper planning of the experiment is essential for all subsequent phases, as mistakes during study planning could lead to irreversible consequences that can potentially invalidate the entire experiment. Furthermore, a clear reporting of all the phases of the experiment, from the design to randomization and analysis, with sufficient details about the methods used, is fundamental to make the research reproducible and to adequately evaluate it. This could be helpful to understand exactly each step and possibly adjust the final statistical analysis, for example, if data were not opportunely analyzed according to the randomization scheme adopted.

### 2.3. Refinement

Russell and Burch indicated that refining the experimental procedures involves not only looking after the animal welfare during the experiment, but also improving the animal’s quality of life during all the procedures it is subjected to during its life in captivity [1]. Starting from this concept, Buchanan-Smith [7] proposed a new definition of refinement: “any approach which avoids or minimises the actual or potential pain, distress and other adverse effects experienced at any time during the life of the animals involved, and which enhances their well-being”. It is important to notice that well-being is not simply the absence of discomfort, but implies a necessary, active and continuous effort for the improvement of the experimental animal’s state. It should be considered that distress may manifest both behaviorally (e.g., overt escape behaviors, approach–avoidance preferences) and physiologically (e.g., movement, vocalization, changes in electroencephalographic activity, heart rate, sympathetic nervous system activity, hypothalamic–pituitary axis activity) [32].

The use of environmental enrichment, which is now explicitly contemplated by the new European Directive 2010/63 [3], is a proven way to provide the animal with greater control of the environment and stimulate the manifestation of behaviors that are inherent to the ecology and etiology of the species used [33]. Regarding the use of environmental enrichments, it is generally thought that the living conditions of animals in captivity are better if they are given the opportunity to express behaviors observed in natural conditions. However, while this is a significant point of view, this is not always the case. In fact, considering that part of the behavioral repertoire of a species can be modified by contingent environmental conditions, and bearing in mind their behavioral flexibility, it follows that animals in captivity can be different from wild ones that live in the environment of origin. The behavioral needs of a captive specimen can therefore be expected to be somewhat different from those of a wild animal. Environmental enrichment must therefore be tailored to each individual situation in order for it to be truly effective. Housing conditions, for example, can significantly improve the welfare of research animals such as rodents, including the provision of shelters and nesting material without neglecting the rather obvious free access to water and food [34]. In addition, all potentially distressing factors should be minimized, such as the noise of ventilation systems. The environment temperature should also be adapted to the physiology of the rodents. If these behavioral needs are not met, the animals can suffer psychophysical stress, which can also compromise the outcome of the experiment. Therefore, improvement strategies do not only improve the welfare of the animals used in research, but also improve the quality and reproducibility of scientific evidence in terms of physical, physiological and ethological needs. Accordingly, enrichment programs must be (i) financially sustainable, (ii) shared with researchers, (iii) established taking into account the time commitment that their implementation requires from the animal facility staff and the fact that they themselves must not interfere with the routine management of animals, as well as (iv) ensure the safety of workers and (v) be monitored with behavioral observations to keep the health status of housed animals under control [35]. Table 1 schematizes the main animal shelters and environmental enrichments.

#### General Health, Well-Being and Anxiety-Like Behavior Evaluation

To assess the general health and well-being of mice and minimize their distress, in accordance with the Refinement principle, it is necessary to measure physiological and behavioral indicators. Over the past three decades, numerous tests has been developed to assess compulsive-like behaviors. In 2001, Roughan and Flecknell [36] studied the possibility that objective behavioral analyses may be used to develop an objective scheme for pain scoring in rats following laparotomy. Starting from 150 behaviors, they selected a set of 16 behaviors that had the greatest value in discriminating treated and control groups.

The Nest Building Test is an important test for assessing the general behavior integrity and well-being of laboratory mice [34,37]. Nest building is a behavior that mice perform for comfort, thermoregulation and for housing their pups; therefore, altered nest building behavior may suggest reduced mouse welfare due to several factors (e.g., thermal stress, general malaise, amount of aggression present within the cage). For example, Hess et al. [38] evaluated the use of naturalistic nesting materials to investigate the potential improvement of the nest quality, concluding that the use of a more naturalistic nesting material allows mice to build more naturalistic nests.

In parallel, the elevated maze test (EPM) and open field test [37,39] can be adopted to test anxiety behavior in mice and evaluate the effectiveness of anxiolytic drugs in neurobiological anxiety research. Briefly, the EPM consists of four arms; two of the opposite arms are walled and the remaining two are open. The amount of time mice spend in the mural arms compared to the open arms in a defined short period provides a measure of anxiety or fear. The test allows researchers to gather information on post-traumatic stress disorder and other conditions characterized by anxious behavior and could be used to screen for new compounds for anxiolytic properties. This test has been used, for example, by Ataka et al. [40] to evaluate the anxiety-like behavior in mice subjected to chronic psychological stress (cPS) and the effects of cPS on the interaction between bone marrow-derived microglia and neurons. Similarly, the Open Field Test measures anxiety-like behavior and locomotor activity [34,37]. The test, which is also used in neurobiological studies, allows researchers to evaluate the basis of anxiety and screening for novel targets and anxiolytic compounds, in addition to the general health and well-being of an animal. Briefly, the test implies the use of a camera to monitor the movement of the animal in and around the peripheral and central areas of a 42 × 42 × 42 cm polyvinyl chloride box. Changes in locomotion may be indicative of altered neurological processes and may therefore reflect abnormal brain function. This test was one of those used by Gouveia and L. Hurst [41] to assess the impact of handling on stress and anxiety in laboratory animals. To complement standard behavioral tests, Home Cage-Monitoring Systems (HCMS) could also be used to assess the general locomotor activity levels and animals’ anxiety as well as investigate the pain response [42]. This method, which allows the prolonged and unbiased observations of spontaneous behavior, has been used by Roughan et al. [43] and Radaelli et al. [44]. Roughan and colleagues [43] evaluated the precision of HCS relative to an experienced human observer in differentiating between the pre- and postoperative behavior of groups of mice undergoing anesthesia and administered different doses of an analgesic; they provided evidence about the powerful role of HCS as a tool for investigating pain responses and analgesic effects following various different types of surgery and other potentially painful conditions in mice, and eventually other rodent species. Radaelli and colleagues [44] adopted the HCS as a complement tool to measure the effect of a brightly lit enclosed chamber (R&L) on mice, concluding that R&L lowered normal walking frequency and likely posed a risk of low-grade neuro-inflammation.

Besides the aforementioned behavior-based methods, physiological methods have been introduced to objectively evaluate the animal experience. Mayer and colleagues [32] reviewed and discussed the approaches for evaluating stress in animals using physiological methods, with emphasis on the transition between the conscious and unconscious states.

## 3. Directive 2010/63/EU—Protection of Animals Used for Scientific Purposes

The use of animal models according to the 3Rs principle in the European Union is regulated by the Directive 2010/63/EU [3]. It is made up of 66 articles and 8 annexes aiming to ensure and adapt scientific and technological progress in the European legislation, which until then was represented by the Council Directive of 24 November 1986 (86/609/EEC [2]). Each state must transpose the Directive 2010/63/EU on the protection of animals used for scientific purposes, and Italy, specifically, must adhere to Legislative Decree 26/2014 [4]. In the 24 years that have passed between the two directives, the acquaintances in the laboratory animal science sector have expanded significantly; it turned out that an update also at the legislative level is therefore essential. In fact, the directive is not a series of rules that favor or prohibit the use of animals in research, but that protect animals used in research laboratories. The primary purpose of the directive is to provide specifications that are as detailed as possible in order to reduce the disparity between member states on methodologies that guarantee the welfare of laboratory animals, making the European landscape more uniform. “Animal Welfare” is in fact a value of the European Union, as described in Article 13 of the Treaty on the Functioning of the European Union [45].

One of the main updates introduced in this European directive with respect to the previous Directive 86/609/EEC is the evaluation of suffering. According to Annex VIII, “The severity of the procedure is determined based on the level of pain, suffering, distress or prolonged damage to which the individual animal is presumably subjected during the procedure itself” [3]. Section I of Annex VIII establishes and defines four categories into which procedures using experimental animals can be divided, as schematized in Table 2.

The assignment of the severity category is based on the risk of the most severe effects, once all the appropriate refinement techniques have been applied. In assigning a procedure to a particular category, the type of procedure itself and other factors such as those summarized in Table 3 are taken into account.

Article 26 of European Regulation 63/2010 provides another strong innovation, “that each breeder, supplier and user sets up an Animal-Welfare Body (AWB)”. Each AWB is composed of at least one person in charge of the welfare and care of animals, a veterinarian and in the case of a structure authorized for experimentation, one scientific member as the guarantor of scientific quality. The figure of the biostatistician within the AWB is still a discussed matter [46]; however, it is crucial for verifying that the study design is compatible with the Reduction principle of the 3Rs.

## 4. Discussion

The widest possible dissemination of information related to the 3Rs is essential if Russell and Burch’s aspiration is to be realized for animal research in the most humane way possible. Although in this commentary we mainly focus on the Reduction principle and the importance of correct study planning and sizing with the most proper experiential design, improvements in both the Replacement and Refinement principles should be considered.

As aforementioned, we are seeing rapid progress in the acceptance of organs-on-a-chip, or, more generally, in the engineering of in vitro models as alternative methods to fulfill the Replacement principle. Increasing evidence supports the potential and utility of these models, for example, for drug/toxicological screening studies [47]. One of the first implementation measures at a legislative level—Commission Implementing Regulation (EU) 2021/1709—concerns the replacement of live animals for the detection of paralytic shellfish poisoning toxins [48]. Similarly, regarding the Refinement principle, improvements to all phases of animal welfare, from housing to analgesia protocols and methods of drug administration, contribute to reduce the suffering of laboratory animals [49]. It is clear that the Reduction principle implies the use of the minimum number of laboratory animals to achieve scientific objectives through the adoption of appropriate study design and statistical methods. Thus, each of the three Rs plays a crucial role in experimental achievement, and they could interact positively or negatively. Indeed, some approaches could simultaneously enhance more than one objective of the 3Rs, or, in other cases, generate contrary effects on two different objectives of the 3Rs. Some examples of positive and negative interactions between the three principles are reported by de Boo et al. [50] and those related to management issues. The harmonization of protocols interacts positively with Replacement and Reduction, the use of in vitro models acts on both Replacement and Refinement, and the implementation of training programs could be viewed as a powerful action point for Reduction and Refinement. On the other hand, as stated by de Boo et al. [50], the implementation of non-animal methods, which require the comparison with the corresponding original in vivo model, could create a negative interaction between the Reduction and the Replacement principles.

In general, in order to conduct useful research, the goal one wants to achieve and the kind of value the experiment possesses are the first aspects to be clearly defined, along with the choice of the most appropriate animal species to reach the most qualitative outcome. Regarding the latter aspect, as reported by Azkona et al. [47], the selection of the species for an animal model should be made to completely resemble the disease completely and to allow a translation of results to humans. Although the choice may be influenced by practical constraints or unscientific reasons, the selection of a species for an animal model requires a multidisciplinary team of specialists who take into account not only financial feasibility, but also biological characteristics, available imaging and molecular techniques, results of previous experiments and even ethical issues for a given species [47].

It should be mentioned that it is possible that discrepancies from the initial experimental project may occur during animal experiment due to some inconveniences. The onset of difficult-to-solve organizational and managerial problems during the implementation phase can lead the researchers to introduce alterations to the original experimental design that affect the subsequent data analysis. In order to avoid invalidating the entire experiment or continuing it with non-robust results, biostatistical support should be required to modify and possibly re-plan an experimental project. This also highlights the importance of a continuous interaction between scientists, managers of facilities and technical personnel, a practice that is a fundamental feature which has become known as the “culture of care”. This embodies the commitment to improve animal welfare, scientific quality, personal care and transparency for all interested stakeholders.

Finally, it is important to emphasize that just as the randomization methods, limitations and potential sources of bias of the studies should be declared in the published manuscript following the accepted guidelines that define the standards of reporting the results of animal research (i.e., Animal Research: Reporting of In Vivo Experiments, ARRIVE) [51], the same details should be adequately cited in the research project submitted to the AWB, in order to have—during study planning—a complete picture of all the implementation phases of the study. Because animal design is a delicate ecosystem, all components and scientists must cooperate with each other. In this view, training/education programs, as well as data/protocols sharing are aspects that should be continuously supported. The EU Reference Laboratory for alternatives to animal testing contributes to the development and testing new animal-free methods to be applied in an integrated safety assessment of chemicals, as well as provides informatics tools and databases to support this [52]. It also promotes the dissemination of information and sharing of knowledge on the 3Rs. Moreover, the “Refinement Database” [53] that collects newly published scientific contributions related to improving or safeguarding the welfare of animals used in research could be a useful initiative in this direction.

## 5. Conclusions

All experimental projects come with trade-offs. It is an ability to conduct animal testing while ensuring the maximum probability of success, maintaining high standards of scientific rigor and accepting practical limits and safeguarding ethics, a skill that constantly requires development. This research field is currently struggling with a high amount of low-power studies, which generate little reliable information. Therefore, a figure such as a biostatistician is needed, able to move and create links in distant fields ranging from the sampling of the population to the ethical aspects of research, for the purpose of valid statistical design, management, analysis and interpretation.

The objectives of harmonization between EU member states, hoped for by the directive, are still a long way off. There are still many gaps to fill before the high standard of animal welfare set by the Union can be achieved [54], and the effort of all the member states is required not only for animal welfare, but for better science and economic reasons [55].

## Figures and Tables

**Table 1:** Animal shelters and environmental enrichments.

Type of Improvements	Description
Hygienic	The environment must be free of insects and parasites and easy to clean and disinfect. In the environment, there must be a rest area, equipped with litter or other soft, dry and clean material. The surfaces on which the animals move must be as dry and clean as possible, so as to ensure the comfort of the animal during movement, prevent slipping and avoid the onset of foot injuries.
Environmental	The temperatures in the housing areas must be set according to the species, the age of the animals and the density of the same in order to avoid situations of heat or cold stress. Ventilation serves to ensure high air quality, low dust and helps to keep the environment at a constant temperature and humidity as much as possible. Finally, air quality should be measured regularly, evaluating the levels of carbon dioxide and oxygen present. Too low humidity causes pathologies in rodents (for example, ringtail) and excessive heat loss. Too high humidity, on the other hand, could favor the development of ammonia in the premises and in the cages. Relative humidity must therefore be managed according to the ambient temperature.
Housing	The animal should be able to organize the space according to its ethological needs; for this reason, the environment should be equipped with material for the construction of nests, adequate litter, perches or platforms. It is useful to populate the environment with objects that allow the animal to carry out the exploratory behavior of the species. The environment must provide complex stimuli that engage the animal in the search for the most appropriate responses, expanding the possibility of expression of his behavioral repertoire. An inadequate housing environment could not guarantee the animal’s well-being and cause psychophysical discomfort (e.g., induce the development of behavioral atypia, stereotypes, and excessive aggression).
Sensory	If the experimental protocol does not allow the interaction with conspecifics, it is essential that they can at least enjoy indirect social contact (i.e., visual, auditory and olfactory). This support develops positive effects on the cognitive abilities of animals, reduces stress during experimental procedures and ensures better interaction between animals and staff or researchers.
Social	Animals need social interaction with conspecifics and therefore should always be housed in pairs or in groups that are as stable and as harmonious as possible, but can also involve negative and stressful aspects such as the aggression of dominant subjects over subordinates.

**Table 2:** Level of pain.

Level of Pain	Description
Non-awakening	Procedures conducted entirely under general anesthesia from which the animal cannot regain consciousness.
Mild	Procedures that are likely to cause mild and short-lived pain, suffering or distress, as well as procedures that do not cause a significant deterioration in the welfare or general condition of the animals.
Moderate	Animal procedures that are likely to cause moderate and short-term pain, suffering or distress, or mild and long-lasting pain, suffering or distress, as well as procedures that are likely to cause moderate deterioration of the welfare or general condition of the animals.
Severe	Procedures on animals that are likely to cause intense pain, suffering or distress, or moderate and long-lasting pain, suffering or distress, as well as procedures that are likely to cause serious deterioration in the welfare or general condition of the animals.

**Table 3:** Factors related to the procedure.

Factors
Nature of the pain, suffering, distress or prolonged harm caused by the Procedure (in all its elements) and relative intensity
Duration, frequency and multiplicity of the techniques used
Cumulative suffering under the procedure
Impediment of natural behavior, due among other things to limitations of the rules in Housing, breeding and care
Type of species and genotype
Maturity, age and sex of the animal
Training experience of the animal with respect to the procedure
Actual severity of the previous procedures, if the animal is intended to be reused
Methods used to reduce or eliminate pain, suffering and distress, including the Improvement of housing, breeding and care conditions

## Data Availability

Not applicable.
